# Risk factors and experiences of prepartum depression in urban- low-income settlement Nairobi Kenya: a mixed-method study

**DOI:** 10.12688/f1000research.27434.3

**Published:** 2021-06-10

**Authors:** Beatrice A. Madeghe, Wambui Kogi-Makau, Sophia Ngala, Manasi Kumar

**Affiliations:** 1 Department of Food Science Nutrition and Technology, University of Nairobi, Nairobi, 00625, Kenya; 2 Department of Psychiatry, College of Health Sciences, University of Nairobi, Nairobi, 00100, Kenya

**Keywords:** Maternal, depression, risk factors, experiences, Kenya

## Abstract

**Background:** Prepartum depression is common among pregnant women and has not been studied much in low and middle-income countries. Evidence shows that mental illnesses are prevalent in urban than in rural areas. The study objective was to determine the magnitude of prepartum depression, risk factors, and real-life experiences of depression among pregnant women.

**Method: **A mixed-method cross-sectional study was conducted. It included 262 pregnant women attending antenatal clinics in two public health facilities in urban low-income settlement Nairobi, Kenya. Edinburgh Postnatal Depression Scale (EPDS) with cut-off >13 was used to classify clinical depressive illness. Further, a focus group discussion was conducted with 20 women identified with depression. Univariable analysis with Odd's Ratio was used to test associations. Variables with a p<0.05 in multivariable regression were considered significant.

**Result: **Out of the 262 women, 33.6% were found to have clinical depression as indicated by EPDS score of >13. Women's gestational age was statistically significantly associated with prepartum depression [OR 4.27 (95% C.I. 2.08 - 8.79), *p* *<* *0.001*]. Income level ≤ 5000 KES was statistically significantly associated with prepartum depression [OR 3.64 (95% C.I.1.25 -10.60), *p=0.018*]. Further, thematic analysis of qualitative indicated that poverty, lack of social support, domestic violence, and unfriendly health care were major contributors to prepartum depression.

**Conclusion: **Significant numbers of pregnant women were found to experience depression. This prevalence rate indicates a high disease burden of women who live with depression, which is not diagnosed because screening of depression is not done in primary health care centers. This study calls for a need and consideration for screening for perinatal depression in primary health care facilities, mainly in resource-poor areas. Interventions targeting means of resolving conflicts in families are highly needed. Such steps would help achieve key sustainable development goals where maternal and child health remains key priority.

## Introduction

Prepartum depression has been identified as a serious health problem but is a neglected component of care for women during pregnancy ^1– 3^. While pregnancy is expected to be the most unique and happiest moments in a woman's life, to some women there is a different scenario filled with tears, fears, and confusion, including stress to severe depression ^4, 5^. Perinatal depression refers to mood disorders during pregnancy and/or after delivery within the first twelve months postpartum; thus, prepartum depression is a mood disorder during pregnancy ^6, 7^. Global prevalence indicates that at least one in ten women in developed countries and two in five women in the developing world develop depression during pregnancy ^8^. The prevalence rates of prepartum depression range between 12.5–42% of women screened for depression symptoms in low and lower middle-income countries ^9, 10^. Systematic reviews that determined the occurrence and determinants of prepartum depression found prevalence rates that range from 15% to 65% globally ^4, 11^. In Kenya, prevalence estimates of depression are found at 32.9% among pregnant adolescents ^12^, with postpartum depression prevalence rate of 13% ^13^.

Research evidence reveals that the stresses of life, especially around pregnancy and childbirth, can affect the emotions of women and behavior of many mothers, hence increasing the risk of depression during pregnancy and after childbirth ^14^. The socio-determinants of health, which refers to the social conditions in which people are born, grow, live, work, and age, influences their health outcomes ^15, 16^. During pregnancy, maternal mental health is fundamental to the health of the mother and the infant's brain health and development; indeed require prioritization early in life course to prevent mental health problems later in life ^1^. Given this backdrop, this study aims to determine the magnitude of prepartum depression, and identify risk factors for prepartum depression, and picture real-life experiences of pregnant women of low-urban income in Nairobi, the capital city of Kenya.

## Methods

### Study setting and participants

This was a mixed-method cross-sectional study that involved pregnant women who attended antenatal care clinics, during the study period March to June 2019. Ethical approval was obtained from The Kenyatta National Hospital/University of Nairobi Ethical and Research Committee (KNH/UoN-ERC Ref: P56/02/2018). The study took place in two public health facilities in two urban low-income settlements, namely Kangemi and Kawangware. The two sites are both growing informal settlements, located on the outskirts of the Nairobi city center. They were chosen purposively because the antenatal clinics (ANC) at these health facilities receive a high volume of pregnant women. Eligibility criteria included all pregnant women aged 15 to 49 years who visited ANCs for antenatal check-up in Kawangware or Kangemi composed the study population. Women who gave a written informed consent and women who are not mentally disturbed to the extent of not giving correct information. 

This cross-sectional study was part of the baseline assessment of a longitudinal cohort study that targeted depressed pregnant women for intervention for depression care among pregnant women in urban low-income settlement in Nairobi Kenya. Sample size was calculated using sample size estimation for longitudinal designs with attrition (Diggle et al., 2002). We needed approximately 100 subjects. To obtain the above sample size at baseline, consecutive sampling was used where every pregnant woman who came for checkup during the study period was asked to be assessed for depression; hence 262 pregnant women were recruited in this study.

### Data collection

***Quantitative data.*** Data were obtained from pregnant women by means of an interview administered questionnaire. All women who came to the ANCs and were queuing in the waiting room were approached to participate. The study purpose was explained to them that participation was voluntary, and refusal to participate will involve no penalty of benefits to which one is entitled at the clinic. The participants were assured about their privacy and confidentiality. After obtaining a written informed consent signed by the respondent based on willingness to participate, the interview commenced.

**Socio-demographic characteristics questionnaire:** Using a questionnaire, pregnant women provided data on socio-demographic characteristics, age, marital status, maternal education level, employment status, partner's occupation, monetary decision-making, and family monthly income.

**Maternal depression questionnaire:** Edinburgh Postnatal Depression Scale (EPDS) was used to assess prepartum depression. The EPDS is a 10-item questionnaire in which women report on how they have been feeling in the past seven days; according to EPDS, a Score of 10 or greater indicates possible depression ^17^. EPDS has a Kiswahili translation version, has been validated for detecting depression in both prepartum and postpartum mothers in many countries, including Kenya ^18^. EPDS is one of the most well-known and evaluated instruments for maternal depression and has demonstrated acceptable clinical utility as a screening tool. It has a sensitivity of 86%, specificity of 78% and a positive predictive value of 73% ^19, 20^. The depression scores were categorized into two levels; EPDS score cut-off score ≥13 indicated existence of depression ^17^.

***Qualitative data.*** Focus Group Discussions (FDGs) were conducted with women who were identified as depressed, in order to get a deeper understanding of women's experiences during the present pregnancy. The non-depressed women were not included in this study because the study interest was to offer an intervention for the depression so there was a need to understand the experiences of depression pregnant women to provide way forward for an intervention study. After the identification of women with depression using EPDS), we asked the pregnant women who were willing to participate in the FDG’s. Convenience sampling was used to select and invited the depressed pregnant women for FGD’s.

The aim of FGD’s was to identify the causes of depression among pregnant women and the experiences around them. In this qualitative inquiry, 20 pregnant women with depressive symptoms (EPDS score cut-off score ≥13) were invited to participate. Two FGDs with a group of 10 participants were conducted from each site of Kangemi and Kawangware, respectively. Face to face discussion was conducted away from the health center by two female field researchers (BM and a research assistant) in a hired hall convenient for the women to discuss. BM has Master in Public Health, and the research assistant has MSc in Clinical Psychology. They were both trained on mental health quantitative and qualitative research methods. The relationship between researcher and pregnant women was established prior to the FDG’s. The FGD’s lasted for 60–90 minutes, and it was conducted in Kiswahili language and was audio-recorded. The interview guide can be found in *extended data* ^21^.

### Data analysis

***Quantitative analysis.*** The filled questionnaires were checked for completeness, errors, and discrepancies; this was followed by data entry, cleaning, and analysis using SPSS version 22. Descriptive statistics such as percentage means and standard deviation were used to summarize the socio-demographic data. Independent variables were categorized and analyzed; the association between independent predictor and outcome variable, using a univariable analysis odds ratio (OR) with 95% confidence interval (CI) was calculated. Those variables that were associated with p<0.001 in the univariable analysis were entered into multivariable logistic regression analysis. Variables with p<0.05 in the multivariable analysis were considered to be significant.

***Qualitative analysis.*** The FDGs in Kiswahili was transcribed and translated into English at the same time. Thematic analysis was employed to process the data. Two coders (BM and a research assistant) were involved in the coding process. The study primarily followed the inductive approach and identified the emerging codes, and to some extent, the deductive approach was also used to determine the codes ^22^. Coding was manually done, the data was coded until all the information required for the study was exhausted, and finally, four major themes emerged from the discussion. COREQ (Consolidated criteria for Reporting Qualitative research) checklist was followed. Informed consent was obtained from participants in this research for future uses of data, such as publication, preservation, and long-term use of research data. Confidentiality was assured. The information collected was kept confidential. Serial numbers were used instead of a name.

## Results

### Socio-demographic characteristics of pregnant women in the urban low-income settlement

All approached pregnant women who attended prenatal clinic during the study period at Kawangware and Kangemi Health center, were screened for depression as part of service delivery. A total of 262 women responded to the questionnaire, 134 from Kangemi and 128 from Kawangware. The mean (SD) age of the 262 women was 25.3±5.0 years with age range of 18–42 years. The majority of the women (82%) were married, with 29.8% having less than high school education and 14% had tertiary level education. About 79.4% of the women had no employment but depended on their partners or parents, only 20.6% were employed. Slightly less than half of the women (43%) were first-time mothers, while 70% of them were in their second trimester during baseline assessment. Almost all the women (98%) owned a basic personal phone, and slightly less than three quarters owned a television (73%). The mean (SD) income level was 10845.8 Kenyan shillings (KES) per month, and almost half of the women (49.6%) lived on an income less than KES 15,000, equivalent of 150 USD per month, while two-fifths of the women (38.9%) had their husbands make decisions on household finances ( Table 1).

**Table 1.** Socio-demographics characteristics of pregnant women from urban low-income settlements in Nairobi, Kenya.

Variable	Category	Frequency (N=262)	Percentage (%)
Age (years)	18–24	134	51.1
	25–42	128	48.9
Age (years)	Mean (SD)	25.3±5.0	
Marital status	Single/lives alone	48	18.3
	Married/lives with a partner	214	81.7
Gestational age	First trimester	24	9.2
	Second trimester	182	69.5
	Third trimester	56	21.4
Employment	No	208	79.4
	Yes	54	20.6
Education level	Primary and below	78	29.8
	Secondary	148	56.5
	Tertiary	36	13.7
Decision maker	Both	110	42.0
	Husband	102	38.9
	Others	50	19.1
Number of children under 18 years	None	112	42.7
	One	80	30.5
	Two	49	18.7
	Three and above	21	8.0
Income per month (KES)	Mean (SD)	10845.8±5005.6	
Income per month (KES)	≤5000	31	11.8
	5,001–10,000	130	49.6
	10,001–15,000	69	26.3
	≥15,000	32	12.2
Owns mobile	Yes	256	97.7
	No	6	2.3
Owns radio	Yes	213	81.3
	No	49	18.7
Owns TV	Yes	192	73.3
	No	70	26.7
Owns laptop	Yes	25	9.5
	No	237	90.5

### Prevalence of prepartum depression in urban low-income settlements in Nairobi, Kenya

Edinburgh Postnatal Depression Scale (EPDS) guided the scoring system ^17^. EPDS has 10 questions which generates scores of 0 to 30 maximum. The depression scores in this study were categorized into two levels (depressed and not depressed), pregnant women with an EPDS score of ≥13 were categorized as depressed. An EPDS score of >13 pointed clinical depression

**Figure 1.**
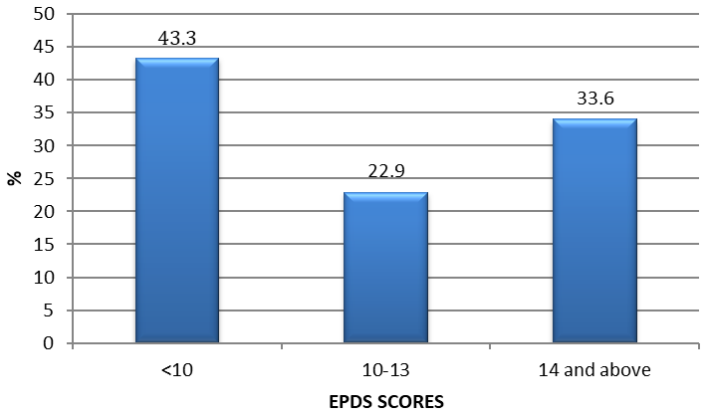
Prepartum depression among pregnant women in urban low income Nairobi -Kenya EPDS (Cutoff >13).

Out of the 262 pregnant women, about a third (33.6%; 95% CI 27.9-40.7) had clinical depression as indicated by EPDS >13 ( Figure 1). Suicidal Ideation (EPDS item 10) revealed that 24% of pregnant women had some suicidal ideation. The mean (SD) EPDS score was 11.1, interquartile range of 10 with a maximum score of 26. All women who appeared to have suicidal ideation and those who were experiencing violence were referred to the mental health counselling services provided at the health facility for treatments; further the second part of the study was an intervention study so they were enrolled to proceed with the intervention.

### Factors associated with prepartum depression

When the socio-demographic characteristics were compared between depressed women and those not depressed, results indicated that women's gestation age in the second trimester (87.5%) was statistically significantly associated with maternal depression [OR 4.27 (95% C.I. 2.08 - 8.79); p< 0.001]. Income levels of ≤ 5000 KES was statistically significantly associated with prepartum depression [OR 3.64; (95% C.I. 1.25 - 10.60); *p = 0.018*] (see Table 2).

**Table 2.** Sociodemographic characteristics associated with prepartum depression in urban low-income settlements Nairobi, Kenya. (EPDS score of ≥13.

Variable	Category	Depression		O.R(95% C.I)	P-Value
		No	Yes		
Age	18–24 Years	74(55.2%)	60(44.8%)	1.08(0.66 to 1.75)	0.768
	25–42 Years	73(57.0%)	55(43.0%)	Ref.	
Marital Status	Lives Alone	26(54.2%)	22(45.8%)	1.10(0.59 to 2.06)	0.764
	Live with a Partner	121(56.5%)	93(43.5%)	Ref.	
Gestational Age	First Trimester	13(54.2%)	11(45.8%)	3.46(1.22 to 9.78)	0.019
	Second Trimester	89(48.9%)	93(51.1%)	4.27(2.08 to 8.79)	**<0.001**
	Third Trimester	45(80.4%)	11(19.6%)	Ref.	
Employment	No	113(54.3%)	95(45.7%)	1.43(0.77 to 2.65)	0.256
	Yes	34(63.0%)	20(37.0%)	Ref.	
Education Level	Primary and below	44(56.4%)	34(43.6%)	1.08(0.49 to 2.41)	0.847
	Secondary	82(55.4%)	66(44.6%)	1.13(0.54 to 2.36)	0.751
	Tertiary	21(58.3%)	15(41.7%)	Ref.	
Decision Maker	Both	71(64.5%)	39(35.5%)	Ref.	
	Husband	53(52.0%)	49(48.0%)	1.68(0.97 to 2.92)	0.064
	Others	23(46.0%)	27(54.0%)	2.14(1.08 to 4.22)	0.029
#children under 18 years	None	58(51.8%)	54(48.2%)	1.24(0.48 to 3.18)	0.652
	One	45(56.3%)	35(43.8%)	1.04(0.39 to 2.74)	0.941
	Two	32(65.3%)	17(34.7%)	0.71(0.25 to 2.01)	0.518
	3 and Above	12(57.1%)	9(42.9%)	Ref.	
Income per month (Ksh.)	≤5000 and Below	14(45.2%)	17(54.8%)	3.64(1.25 to 10.60)	**0.018**
	5,001-10,000	69(53.1%)	61(46.9%)	2.65(1.11 to 6.34)	0.028
	10,001-15,000	40(58.0%)	29(42.0%)	2.17(0.86 to 5.52)	0.102
	≥15,001	24(75.0%)	8(25.0%)	Ref.	
Owns Mobile	Yes	144(56.3%)	112(43.8%)	0.78(0.15 to 3.93)	0.761
	No	3(50.0%)	3(50.0%)	Ref.	
Owns Radio	Yes	123(57.7%)	90(42.3%)	0.70(0.38 to 1.31)	0.266
	No	24(49.0%)	25(51.0%)	Ref.	
Owns TV	Yes	118(61.5%)	74(38.5%)	0.44(0.25 to 0.77)	0.004
	No	29(41.4%)	41(58.6%)	Ref.	
Owns Laptop	Yes	18(72.0%)	7(28.0%)	0.46(0.19 to 1.15)	0.099
	No	129(54.4%)	108(45.6%)	Ref.	
Household size	Mean±SD	3.0±1.2	2.8±1.2	0.83(0.67 to 1.02)	0.075

### Qualitative results

Focus Group Discussions included pregnant women with age range of 18 to 33, and EPDS score range of 13–25 and were in their second trimesters. Table 3 provides participant's characteristics in the FGDs.

**Table 3.** Characteristics of the participants in focus group discussions among pregnant women in urban low-income settlements in Nairobi, Kenya.

Participant ID	Age (years)	Marital status	Education	No. of children	Gestational age	EPDS
**Depressed women from Kawangware**						
1	19	Single	Secondary	1	6	14
2	22	Married	Primary	1	4	18
3	26	Married	Primary	0	5	17
4	23	Married	Secondary	0	6	16
5	20	Married	Primary	1	5	14
6	21	Married	Secondary	0	5	15
7	18	Married	Primary	0	5	14
8	30	Single	Collage	2	6	16
9	24	Married	Secondary	0	6	14
10	33	Married	Secondary	1	6	19
**Depressed women from Kangemi**						
11	23	Married	Primary	1	5	14
12	22	Single	Secondary	0	6	25
13	25	Married	Secondary	0	6	18
14	22	Married	Secondary	0	6	17
15	30	Married	College	0	5	14
16	21	Married	Primary	0	4	16
17	33	Married	Primary	3	5	17
18	23	Married	Primary	1	6	14
19	24	Single	Secondary	0	5	19
20	20	Married	Secondary	2	4	16

The qualitative results from the FDGs were categorized into four major themes: **Povert**y- which manifested through a financial struggle, unemployment and food insecurity; **Social support** - women reported inadequate support from their partners/husbands, the lack of a trustworthy person/friend who they could share their worries, loneliness, living far from family members, and feeling neglected; **Marital disharmony** – issues of domestic violence (physical abuse and emotional abuse), and separation/divorce were reported; **Trauma** experiences - women reported fear and worries about childbirth and birth outcome, considering that most of them were first-time mothers, and some women reported a previous loss of a child, previous birth difficulties, and fear of facing health care providers. Each of these themes is discussed in detail below and experiences in quotes.

**Poverty:** Poverty is the root cause of stress and depression that contributes to other risk factors of depression. Women reported having not enough making and everything seeming difficult because of this. The three subthemes identified were financial struggles, food insecurity, and lack of employment. The experiences are explained in quotes below:-

***Financial struggles***

Pregnant women reported having financial struggles because they have no income, or their partner not having any employment, so it becomes so difficult for them to do things.

*"I don't have a job, and I'm pregnant, and my husband doesn't have a job, and we live that way looking here and there; we are supposed to pay rent, food, and different kinds of needs. So you live with questions and start questioning yourself."* Participant 20

*"Lack of money is a big problem; if I have money in my pocket, I would be just fine."* Participant 15

*"Because I have no job, I’m only a stay home mom, when I lack something and I ask my husband, and he doesn’t have money he become harsh, then it results in quarreling, just that."* Participant 5

***Food insecurity***

Some pregnant women reported a lack of enough food, or sometimes basic food may be available, but the food that they crave during their pregnancy they cannot get.

*“ Sometimes we don’t have food, and there is nothing to eat this causes me to be sad."* Participant 12

*"My husband will leave me and there is nothing to eat at home I'm pregnant, and I have no job so that one usually bores me so much."* Participant 17

*“Sometimes the food may be available but my husband stresses me, how will I be able to eat? You cannot."* Participant 10

*"Sometimes the basic food is available, but the food that I crave/want to eat I can't get because I don’t have money, I love and admire eating pizza, but there is not enough money to buy it."* Participant 11

*"When I ask my husband about house needs, he becomes harsh and doesn't understand me."* Participant 6

**Social support:** Most of the pregnant women felt that they lack emotional and financial support from the partners responsible for their pregnancy. In addition, some of the partners rejected the pregnancy, and don’t want to take any responsibilities.

*"I stay with my husband's family, and there is no any kind of support they offer me, and my husband himself doesn't support me, so the people who support me are neighbors, when I have a problem I tell them and they offer me some support.”* Participant 2

*"Before I became pregnant, my boyfriend wanted me to be pregnant and have a baby and that he will marry me. But when I became pregnant he doesn’t want to marry me, he doesn’t do what he said, that makes very sad.."* Participant 4

*"I was admitted in the hospital and I was alone, the doctors called my boyfriend because there was a need for me to get blood transfusion; they called him to come to replace the blood. He doesn’t know me.’ He just talks rubbish."* Participant 12

*"As for me, my boyfriend doesn’t want to support me, so I’m prepared to give birth and raise the baby myself because he doesn’t want to know about me and baby"* Participant 19

*"My husband doesn’t care when he sees me cry or sad, he will ask me, 'what are you crying for? Just cry when you are tired you keep quiet".* Participant 5

Some women reported that they don’t have a trusted person around them who they can share their worries and talk heart to heart. Life conditions in slums where people live in one plot, some neighbors are stubborn and like making fun to others, contributing to stress, anger and depression. Some women stay away from their family members, and so they feel they lack support.

*“I’m all alone; I don't have anybody around that I can trust to the level that I can share my story."* Participant 7

*“I have some people around me, but I have never trusted somebody so much, because some of them cannot be true."* Participant 5

*"I cannot trust someone just like that; some people, you can tell them your problems thinking that it’s only between you two, but after that you will hear it with everybody, and that is very embarrassing."* Participant 15

*“There are some people whom you can tell and maybe you feel that she is a friend, you sit with her and tell her; she also supports you as if she has pity over you, but maybe after you leave she is a type that laughs at you."* Participant 16

***Marital disharmony***. In the FDGs, women reported hopelessness and helplessness around the experience of domestic abuse, which manifested as both physical and emotional abuse. The experiences that women go through have been narrated below:-

***Physical abuse***

*"One day, my husband came and started quarreling with me, and then he later slapped me, and then pushed me. I fell on the table and hit my stomach. I started feeling pain on the stomach and thought my baby in my belly was hurt."* Participant 6

*"My husband has a lot of disrespect whereby he sometimes spends a night out when he comes back he doesn’t say anything, when I ask him where he was then at that point we start a big fight, and he will leave without leaving you food or any money, usually that bores me so much."* Participant 17

Some women defend themselves.

*"Yes; whenever he starts, we just hit each other, there is no sparing him; so he also fears me, I have a lot of energy, and he cannot quarrel with me so much."* Participant 14

Sometimes women are forced to stay with abusive partners even if they are beaten, they can’t go anywhere because they can’t sustain themselves.

*"If I go tell my parents they say you cannot live a life with a husband without being beaten"* Participant 3

*“Now if you leave your partner, where will you go?"* Participant 9

*"I don't have a job; and I don't have money; if I had a job and money would get myself out. Where will I go to stay*?" Participant 11

***Psychological abuse***

Women reported being psychologically abused, by their partners who refuses to offer emotional support to their wives.

*"My husband is keeping quiet, if I don't talk, he cannot talk to me and this hurts me so much."* Participant 19

*"Mine says that he will beat me, and that he is not the one responsible for the pregnancy, then he says that I should abort, so I abandoned him."* Participant 13

*"Mine also doesn't want to talk, when I’m tired and tell him that today I am not feeling well’, he tells me ‘it's up to you’.* Participant 9

***Separation/Divorce***

Some women experienced separation or divorce.

*"I don't stay with him because he is so contemptuous; he cannot even offer me any support, he has even blacklisted my number."* Participant 12

*"I don't know how to put it, but we parted ways those days when I was the first month, so all along I have been surviving, and I have become used to it."* Participant 8

***Trauma experiences.*** Some women had previous trauma experiences, such as loss of a child and or difficult birth experiences.

*"Fear of how it will be the day I go for delivery I don't know if my baby will be alive or die, so I have so much thought and think a lot"* Participant 3

*"I fear how that situation will be during childbirth, so my mind is being tormented thinking the day that I will go to deliver."* Participant 7

*"I fear delivery, and that is my stress, since I have never had a baby before, people tell me it is fine but it frightens me"* Participant 1

Some women feel that health care is unfriendly. When they go to the clinic, nothing is explained to them during a physical examination. For example, pregnant women are not given any feedback about how they are doing, or if their babies are growing well. They reported that they are dismissed without any information given to them, only the date to come back for checkup is provided. Therefore, the women reported that health care it-self sometimes is a source of stress.

*"They pressed my belly so hard and painful, and they tell me go you are done, and another one is called; they don’t explain anything well, I don’t know how my baby is doing, so I don't understand."* Participant 6

## Discussion

In our study, 33.6% of pregnant women in the study had clinical depression. This prevalence rate indicates a high number of women who live with depression and calls for interventions to support women with this conditions. This estimate lies within the wide range of prior prepartum prevalence rates of depressive symptoms with range of (12.5–42%) among pregnant women in low and middle-income countries ^9, 10^. Our findings revealed relatively similar prevalence rates to the study by De Oliveira *et al*. ^11^, who found 37.5% depression prevalence among Hispanic pregnant women in South Florida using PHQ-9. A study by Sheeba *et al*. ^23^ in Ethiopia found a prevalence rate of 35.7% among pregnant women using EPDS >13. Likewise, among Chinese women a prevalence rate of 28.5% was found among pregnant women in late pregnancy using the Self-rating Depression Scale ^24^. A study by Shrestha ^20^ from Nepal reported a point prevalence of 18% among pregnant women using EPDS ≥10. Another study in Ethiopia by Duko *et al*. ^25^ found a prevalence rate of 21.5% among prenatal mothers using EPDS ≥13.

Various prevalence estimates of prenatal depression have been reported in various countries, and prepartum depression has been experienced differently across countries. The differences in estimates could be due to methodological differences in the ways the studies were conducted, or the settings where the studies were conducted; the timing of pregnancy, screening instruments used, and the cut-off values used to classify mothers as depressed as described in the literature see reviews ^16, 25– 27^. However, despite all those differences, the most important thing noteworthy is that all the studies show significant levels of depression during pregnancy that calls for intervention.

In our study the factors that were associated with prepartum depression included lower income levels and gestational age; specifically women in the second trimester these two factors were significantly statistically associated with prepartum depression. Furthermore, the qualitative findings revealed poverty to be the primary determinant of prepartum depression, especially financial struggles due to lack of stable income and food insecurity. In this study, most of the study participants were young women who belong to the low-income group, and most of them were first-time mothers. These women were unemployed and were entirely dependent on their spouses or partners and family members. It is hypothesized that low income increases the likelihood of poor living conditions; financial struggle influences personal relationships, leading to psychosocial stress. This hypothesis agrees with our findings where poverty, including financial struggles and food insecurity, were associated with prepartum depression. Additionally, the qualitative findings revealed other factors such as marital disharmony, where women experienced domestic violence, divorce or separation. Other factors included lack of socio- support, and trauma experiences to be among factors that cause pregnant women to be depressed

Our results have similarities with other studies in the literature, which identified risk factors for prepartum depression including maternal age, socio-economic status ^15^, domestic violence, social support, history of previous mental disorder ^23^, and pregnancy-related complications ^16^. The study by Shrestha in Nepal reported higher odds for health problems, gestational age, sex preference, and spousal alcohol intake to be associated with depression ^20^; similar to our study the gestational age was associated with prepartum depression. Another study by Sheeba *et al*. in Ethiopia ^23^ reported that age group, educational qualification and occupation were significant predictors of prenatal depression, and socio-economic status was not significantly associated with depression; these results contrast with our findings where age, education level, and occupation were not related to prepartum depression.

The risk factors related to prepartum depression may not be similar across countries. Some studies may find similar risk factors associated with prepartum depression and other studies may not have found similar factors associated with depression ^16, 25, 26^. This could be attributed due to cultural differences, population differences, and study setting, and living conditions that cause people to experience different risk factors for prepartum depression.

### Strengths and limitations

This study used mixed methods, both quantitative and qualitative. The qualitative part was useful in gaining a better understanding of the experiences of prepartum depression and has complemented the quantitative part of the study providing a full story. Also prepartum depression has not been studied much in low and middle-income countries, and is contributing to a high disease burden. Therefore, our study focus on prepartum depression contributes to filling a knowledge gap and awareness to health-care providers, researchers, policymakers, and the public about the high rates and risk factors of prepartum depression. However, due to reasons that the recruitment occurred at the ANC where the attendance rate is not 100% for all residents in the settlements, many women with depressive symptoms may not been reached, and even those who came to the ANC, not all of them were screened leading to underreporting of the cases hence the result cannot be generalized. Moreover, although EPDS is an established and widely used screening tool for maternal depression with high speciﬁcity and sensitivity, it is not as a definitive diagnosis, hence the inherent limitation of using this tool. Also other possible confounding factors such as child sex preference, history of previous mental disorder, spousal alcohol intake as a risk factor for depression were not assessed hence limitation of this study. Additionally this was a cross sectional study and as a limitation cross sectional studies does not establish the causal relationship hence the limitation for this study

## Conclusion

Considerable numbers of pregnant women, about a third of the women, were found to experience maternal depression in the urban-low income of Nairobi, Kenya. Our study findings indicate many women who live with prepartum depression, which is neither diagnosed nor treated since the ANC do not carry out routine screening for prepartum depression. Therefore, women are suffering without adequate services or timely help. This study calls for urgent consideration of screening of perinatal depression at primary health care facilities so that women can get help through counseling and be provided with social support. We strongly feel that the ANC nurses and primary health care staff also need to be trained in delivering respectful, patient-centered services where the mental health of these vulnerable women is prioritized. Interventions targeting means of resolving conflicts and intimate partner violence are highly needed. This will contribute towards efforts on global mental health and sustainable development in prioritizing perinatal mental health and childhood mental health because acting early in the life course is crucial to preventing mental health problems later.

## Data availability

### Underlying data

Figshare: Socio-demography and Depression Questionnaire, https://doi.org/10.6084/m9.figshare.13265297.v1 ^28^.

Figshare: Maternal Depression experiences FDG transcription, https://doi.org/10.6084/m9.figshare.13265549.v1 ^29^.

### Extended data

Figshare: Maternal Depression Experiences FDGs Interview guide, https://doi.org/10.6084/m9.figshare.13265420.v2 ^21^.

Data are available under the terms of the Creative Commons Attribution 4.0 International license (CC-BY 4.0).
