# Supplementary material for: Risk factors and experiences of prepartum depression in urban- low-income settlement Nairobi Kenya: a mixed-method study
Source: F1000Res. 2021 Jun 10;9:1495. Originally published 2020 Dec 21. [Version 3] doi: 10.12688/f1000research.27434.3 (PMC8207804; doi:10.12688/f1000research.27434.3)
Supplement: Peer review history

# Peer review: Reviewer response for version 3

**DOI:** 10.5256/f1000research.57589.r87287

**Published:** 2021-06-15

**Authors:** Rahul Shidhaye

**Affiliations:** 1 Pravara Institute of Medical Sciences, Ahmednagar, Maharashtra, India

Thank you for undertaking all the revisions. The revised measures of association (odds ratios) now look appropriate. I am satisfied will all the changes.

Is the work clearly and accurately presented and does it cite the current literature?

Partly

If applicable, is the statistical analysis and its interpretation appropriate?

Partly

Are all the source data underlying the results available to ensure full reproducibility?

Yes

Is the study design appropriate and is the work technically sound?

Partly

Are the conclusions drawn adequately supported by the results?

Partly

Are sufficient details of methods and analysis provided to allow replication by others?

Yes

Reviewer Expertise:

Psychiatric epidemiology, perinatal mental health, implementation science

I confirm that I have read this submission and believe that I have an appropriate level of expertise to confirm that it is of an acceptable scientific standard.

# Peer review: Reviewer response for version 2

**DOI:** 10.5256/f1000research.55971.r83411

**Published:** 2021-06-02

**Authors:** Rahul Shidhaye

**Affiliations:** 1 Pravara Institute of Medical Sciences, Ahmednagar, Maharashtra, India

Please check the odds ratio for the association of income levels with prepartum depression. From your results it seems that lower income level is a protective factor and reduces the risk for prepartum depression.

Is the work clearly and accurately presented and does it cite the current literature?

Partly

If applicable, is the statistical analysis and its interpretation appropriate?

Partly

Are all the source data underlying the results available to ensure full reproducibility?

Yes

Is the study design appropriate and is the work technically sound?

Partly

Are the conclusions drawn adequately supported by the results?

Partly

Are sufficient details of methods and analysis provided to allow replication by others?

Yes

Reviewer Expertise:

Psychiatric epidemiology, perinatal mental health, implementation science

I confirm that I have read this submission and believe that I have an appropriate level of expertise to confirm that it is of an acceptable scientific standard, however I have significant reservations, as outlined above.

# Response

**Published:** 2021-06-07

**Authors:** Beatrice Madeghe

**Affiliations:** University of Nairobi, Kenya

Comment: Please check the odds ratio for the association of income levels with prepartum depression. From your results, it seems that lower-income level is a protective factor and reduces the risk for prepartum depression

 Response: Thank you for this observation. I have revised table 2 and corrected the odds ratio for the associations in the table and in the abstract and in the result section.

# Peer review: Reviewer response for version 2

**DOI:** 10.5256/f1000research.55971.r83412

**Published:** 2021-04-30

**Authors:** Irene Inwani

**Affiliations:** 1 Department of Pediatrics, Kenyatta National Hospital, Nairobi, Kenya

The issues raised in the last review have been satisfactorily addressed

Is the work clearly and accurately presented and does it cite the current literature?

Partly

If applicable, is the statistical analysis and its interpretation appropriate?

Partly

Are all the source data underlying the results available to ensure full reproducibility?

Yes

Is the study design appropriate and is the work technically sound?

Yes

Are the conclusions drawn adequately supported by the results?

Partly

Are sufficient details of methods and analysis provided to allow replication by others?

Yes

Reviewer Expertise:

Pediatric, adolescents and maternal health.

I confirm that I have read this submission and believe that I have an appropriate level of expertise to confirm that it is of an acceptable scientific standard.

# Peer review: Reviewer response for version 1

**DOI:** 10.5256/f1000research.30318.r76448

**Published:** 2021-02-04

**Authors:** Rahul Shidhaye

**Affiliations:** 1 Pravara Institute of Medical Sciences, Ahmednagar, Maharashtra, India

In this paper titled, 'Risk factors and experiences of prepartum depression among pregnant women in urban-low income in Nairobi Kenya: a mixed methods study,' Madeghe and colleagues present the prevalence of prepartum depression, risk factors, and real-life experiences of depression among pregnant women. There are few concerns that need to be addressed and some suggestions to further improve this paper.

1. **Title:** It will be better to use the term 'antenatal depression' instead of 'prepartum depression' as the former is more commonly used in the literature. A quick pubmed search returned 609 articles for the term, 'antenatal depression' compared to 9 results when 'prepartum depression' was used. In case you agree with this suggestion, please make the changes throughout the draft. I assume you wish to say urban-low income settlements/setting? Please add an appropriate word in the title. Prepartum depression will be present ONLY in pregnant women. It will be good to say either, 'prepartum/antenatal depression in urban low......' or 'depression among pregnant women in urban low.....'  
2. **Abstract:** It is mentioned, ' *Women's gestational age in the second trimester (87.5%) was statistically significantly associated with prepartum depression (p<0.001).' *Please can you provide the measure of association (odds ratio) to support this statement? It is not clear what 87.5% means. Is it that 87.5% of women in their second trimester had depression? I have the same comment for the next statement in the abstract on income level. 
3. **Sample size:** Please provide the rationale for recruiting 262 pregnant women in this study.
4. It will be good to provide the reference for the cohort study mentioned in the methods section. 
5. Did you use any conceptual framework or prior literature to select the socio-demographic characteristics? A number of factors such as domestic violence, gender preference of the child, and nutritional status of women are not included. Please clarify. 
6. Generally, the cut-off of 11/12 is used for EPDS. Please clarify the reason for choosing 12/13 as the cut-off.
7. The following sentence is not clear, 'The data was coded until when the ability to obtain additional new information was exhausted for the study purpose.'
8. How many women were approached and what proportion of them consented to participate in the study? Please provide these details in the first paragraph of the results. 
9.  Although 33.6% of women had depression, table 2 mentions 174 women as depressed. Please check. 
10. Please provide actual n for each of the variables. 
11. The percentages presented in table 2 make very little sense. The readers will be interested in knowing the proportion of women who screened positive for depression for one level of a variable compared to another level of the same variable. To illustrate this, it will be good to know what proportion of women in the age group of 18-24 screened positive for depression and also the proportion of women in the age group of 25-42 who screened positive for depression. The current numbers do not provide that information. 
12. It seems that lower income level was a protective factor and resulted in lower odds of depression (OR: 0.39, 95% CI 0.23-0.66). This completely contradicts the findings from your qualitative study as well as from the past literature. 
13. Please present the findings related to the univariable association of each of the explanatory variables with the outcome. I assume that the findings in table 2 are that of multivariable association? Please clarify. 
14. The analysis undertaken is simple/univariable and multiple/multivariable logistic regression and not multivariate regression. Please correct. 
  .
15. One of the important limitations is the cross-sectional nature of this study which precludes any causal inferences. This needs to be included in the limitations. 
16. It is also likely to have confounding bias as the data on key explanatory variables is not included. 
17. The use of a screening tool for depression (EPDS) also needs to be included as one of the limitations. 
18. Please read the paper carefully to address the grammatical errors.

Is the work clearly and accurately presented and does it cite the current literature?

Partly

If applicable, is the statistical analysis and its interpretation appropriate?

Partly

Are all the source data underlying the results available to ensure full reproducibility?

Yes

Is the study design appropriate and is the work technically sound?

Partly

Are the conclusions drawn adequately supported by the results?

Partly

Are sufficient details of methods and analysis provided to allow replication by others?

Yes

Reviewer Expertise:

Psychiatric epidemiology, perinatal mental health, implementation science

I confirm that I have read this submission and believe that I have an appropriate level of expertise to state that I do not consider it to be of an acceptable scientific standard, for reasons outlined above.

# Response

**Published:** 2021-04-07

**Authors:** Beatrice Madeghe

**Affiliations:** University of Nairobi, Kenya

**Reviewer 2: Comments and responses**

**1. Title:** It will be better to use the term 'antenatal depression' instead of 'prepartum depression' as the former is more commonly used in the literature. A quick PubMed search returned 609 articles for the term, 'antenatal depression' compared to 9 results when 'prepartum depression' was used. In case you agree with this suggestion, please make the changes throughout the draft. I assume you wish to say urban-low income settlements/setting? Please add an appropriate word in the title. Prepartum depression will be present ONLY in pregnant women. It will be good to say either, 'prepartum/antenatal depression in urban low......' or 'depression among pregnant women in urban low....?

**Responses:-**

 Thank you for this suggestion and it is true pre-partum is not commonly used.  I request to maintain the prepartum term because I have defined it and it has the same meaning as prenatal.  I have maintained it throughout the text. I have structured the title, and I have added the words settlements as advised, now the title read as:-“Risk factors and experiences of prepartum depression in urban-low income settlements Nairobi-Kenya: a mixed-method study” see page 1.

 2. **Abstract:** It is mentioned, ' *Women's gestational age in the second trimester (87.5%) was statistically significantly associated with prepartum depression (p<0.001).' *Please can you provide the measure of association (odds ratio) to support this statement? It is not clear what 87.5% means. Is it that 87.5% of women in their second trimester had depression? I have the same comment for the next statement in the abstract on income level?

**Response: -**

 This was revised.  I have provided the measures of associations to support the statements in the abstract and text as follows:- Women's gestational age was statistically significantly associated with prepartum depression [OR 3.37; (95% C.I 1.60 - 7.10); *p* *<* *0.001*]. Income levels <10,000 KES was statistically significantly associated with prepartum depression [OR 0.39; (95%; C.I 0.23 – 0.66); *p <* *0.001*. See abstract.

 3. **Sample size:** Please provide the rationale for recruiting 262 pregnant women in this study?

**Response: -**

 This was revised and explained in the text. The rationale for recruiting 262 women is because this was the baseline study for the intervention longitudinal study.  So sample size was calculated using Sample size estimation for longitudinal designs with attrition (Diggle et al., 2002). We needed approximately 100 subjects in each group, where 50 would be the intervention and 50 would be the intervention group. Therefore to obtain the above sample of women, we consecutively screened 262 pregnant women at baseline and obtained 100 depressed pregnant women. See method.

 4. It will be good to provide the reference for the cohort study mentioned in the methods section? 

**Response: -**

 Thank you for this suggestion, the intervention results for this study are not yet published and still under review, this was the baseline data that we are sharing, that’s why we are unable to provide the reference cohort.

 5. Did you use any conceptual framework or prior literature to select the socio-demographic characteristics? A number of factors such as domestic violence, gender preference of the child, and nutritional status of women are not included. Please clarify?

**Response: -**

 Thank you for this comment. Yes, the literature guided the process of selecting the socio-demographic factors; in this study, the association between nutritional status and prepartum depression was studied in details, and the nutritional factors/status associated with prepartum depression were evaluated and discussed in another paper, which is still under review, we also conducted the focus group discussion to understanding the real-life experiences of depressed women.  I admit that as a limitation to this study some contributing factors were not included in the sociodemographic questionnaire.  However, in the focus group discussion, domestic violence came up and was captured as contributing factor for depression in this study. This limitation was added as the limitation of to study, that some factors such as child sex preference, spouse alcohol intake were not assessed.

 6. Generally, the cut-off of 11/12 is used for EPDS. Please clarify the reason for choosing 12/13 as the cut-off?

**Response: -**

 I used The **Edinburgh** **Postnatal Depression Scale (EPDS). **According to the scoring system of this tool, a score of 10 or greater indicates possible depression. Mothers who score above 13 are likely to be suffering from clinical depression of varying severity.  The maximum score is 30. ^17^

 Therefore I followed the cut-off guidance provided in this scale where an EPDS, a score of 10 to 13 indicates possible depression of moderate severity, and a score of above 13 indicates clinical depression. In this study, all women who had EPDS SCORE of 10 to 13 were classified with possible depression, and those with EPDS scores of above 13 were classified as having clinical depression. Source: Cox, J.L., Holden, J.M., and Sagovsky, R. 1987.

 7. The following sentence is not clear, 'The data was coded until when the ability to obtain additional new information was exhausted for the study purpose?

**Response:** -

 The sentence was reviewed and made clear. Data was coded until all the information required for the study was exhausted. See FGDs results.

 8. How many women were approached and what proportion of them consented to participate in the study? Please provide these details in the first paragraph of the results? 

**Responses:-**

 This section was revised and added to the text. This cross-sectional study was part of the baseline assessment of a longitudinal cohort study that targeted depressed pregnant women for intervention. Therefore the sample size was calculated using Sample size estimation for longitudinal designs with attrition (Diggle et al., 2002). We needed approximately 100 subjects. To obtain the above sample size at baseline, consecutive sampling was used where every pregnant woman who came for a checkup during the study period was assessed for depression as part of service delivery, hence 262 pregnant women were recruited which enabled a sample of 100 depressed women. See method.

 9. Although 33.6% of women had depression, table 2 mentions 174 women as depressed. Please check?

**Responses: -**

 Thank you for this comment. This was the mistake, the table was not labeled correctly. I have revised this table and the table has been labeled correctly.

 10. Please provide actual n for each of the variables?

**Response: -**

 This has been revised the actual numbers in the table have been provided. See table 2.

 11. The percentages presented in table 2 make very little sense. The readers will be interested in knowing the proportion of women who screened positive for depression for one level of a variable compared to another level of the same variable. To illustrate this, it will be good to know what proportion of women in the age group of 18-24 screened positive for depression and also the proportion of women in the age group of 25-42 who screened positive for depression. The current numbers do not provide that information?

**Response: -**

 This comment has been revised

 I have provided the proportion of women who screened positive for depression for one level of a variable were compared to another level of the same variable. See table2.

 12. It seems that a lower-income level was a protective factor and resulted in lower odds of depression (OR: 0.39, 95% CI 0.23-0.66). This completely contradicts the findings from your qualitative study as well as from the past literature? 

**Response: -**

 This was an error in the labeling of table 2, which caused results to read the opposite, this has been corrected, and the table labeled correctly. The result reads well that the lower-income level is the risk factor for depression. See table 2.

 13. Please present the findings related to the univariable association of each of the explanatory variables with the outcome. I assume that the findings in table 2 are that of multivariable association? Please clarify? 

**Response:-**

 Thank you for this comment, the factors that were significant in the univariable association were the same as in the multivariable association, so I only provided the multivariable table because the factor that was significant was the same. See data analysis.

 14. The analysis undertaken is simple/univariable and multiple/multivariable logistic regression and not multivariate regression. Please correct?

**Response:-**

 Thank you for this correction, I have corrected this error. The analysis undertaken was univariable and multivariable logistic regression. See data analysis.

 15. One of the important limitations is the cross-sectional nature of this study which precludes any causal inferences. This needs to be included in the limitations?

**Response: -**

 Thank you for this comment. This limitation has been added to this study, This study was a cross-sectional study and as a limitation, cross-sectional studies do not establish the causal relationship hence the limitation for this study. See study limitation.

 16. It is also likely to have confounding bias as the data on key explanatory variables is not included? 

**Response: -**

 This section has been revised and added as a limitation to the study that, other possible confounding factors such as child preference, previous history of depression, and partner alcohol intake as a risk factor for depression were not assessed hence a limitation to this study. See study limitation.

 17. The use of a screening tool for depression (EPDS) also needs to be included as one of the limitations?

**Response: -**

 Thank you for this comment, this limitation was added to the text that; Although EPDS is an established and widely used screening tool for maternal depression with high speciﬁcity and sensitivity, it is not as a definitive diagnosis, hence the inherent limitation of using this tool. See study limitation.

 18. Please read the paper carefully to address the grammatical errors?

**Response: -**

 The paper was carefully read through and all possible grammatical errors addressed. Thank you

# Peer review: Reviewer response for version 1

**DOI:** 10.5256/f1000research.30318.r76447

**Published:** 2021-01-25

**Authors:** Irene Inwani

**Affiliations:** 1 Department of Pediatrics, Kenyatta National Hospital, Nairobi, Kenya

This manuscript addresses an important and to some extent neglected area of health care and research. It is a well written manuscript but there are areas that require some improvement as highlighted below:

1. What was the rationale of recruiting women aged 18 to 44 years only when we know age is a predictor of depression?
2. What was the justification for not including a sample from women identified as not depressed in the FGDs to understand if their experiences were similar to the sampled group?
3. Eligibility criteria in this study were only age and pregnancy. What criteria made participants not eligible for the study? Which confounding factors were considered?
4. Which quality assurance measures were put in place for qualitative data analysis?
5. Ethically; what happened to women found to have depression and especially those who had suicidal ideation as well as those experiencing violence?
6. Please check the following:

-
  - Age range in the text is 18-44 while in the tables it is 18-42
  - Table 1; what does the percentage of the mean age represent?
  - Parity is equated to the number of children below 18 years; this is erroneous, a 44 can have children above 18 and therefore be misclassified.
  - In the table the bands for income overlap, check.
  - Confirm the proportion of those earning less than 15000.
  - Revise subheadings to reflect the issue being addressed and leave out the population and settings which are described in the methods.
  - Reported range of depression score conflict some sections 0-30, others 0-26.
  - Table 2; confirm the totals (N) for depressed and non-depressed.
  - Table 3 categorize per site.
  - The qualitative data need to be reworked and triangulated appropriately with the quantitative data. The themes, example poverty and financial struggles are closely related and therefore need to see which quotes fit well in each theme.
  - Review the language in the quotes and generally in the qualitative section.
  - Discussion section; A good discussion but can be improved by focusing on the audience and key message built from the major findings. Check findings from other countries, regions and local which used a similar and different methodology, and if possible similar context.
  - Discuss some more weaknesses/limitations such as cross-sectional nature of the study therefore not able to establish a causal relationship, not having assessed other possible contributors to depression and neonatal outcomes

Is the work clearly and accurately presented and does it cite the current literature?

Partly

If applicable, is the statistical analysis and its interpretation appropriate?

Partly

Are all the source data underlying the results available to ensure full reproducibility?

Yes

Is the study design appropriate and is the work technically sound?

Yes

Are the conclusions drawn adequately supported by the results?

Partly

Are sufficient details of methods and analysis provided to allow replication by others?

Yes

Reviewer Expertise:

Pediatric, adolescents and maternal health.

I confirm that I have read this submission and believe that I have an appropriate level of expertise to confirm that it is of an acceptable scientific standard, however I have significant reservations, as outlined above.

# Response

**Published:** 2021-04-07

**Authors:** Beatrice Madeghe

**Affiliations:** University of Nairobi, Kenya

**Reviewer 1: Comments and responses**

 1. What was the rationale of recruiting women aged 18 to 44 years only when we know age is a predictor of depression **?**

**Response: -** Thank you for this comment.  This was a mistake and has been corrected in the text.  The study participants included Women of reproductive age of 15-49 in Kenya. The actual age range of participants who were present and recruited in the study and their data analyzed had an age range of 18 to 42. This has been corrected in the text. See method, Study participants.

 2. What was the justification for not including a sample from women identified as not depressed in the FGDs to understand if their experiences were similar to the sampled group?

**Responses: **Thank you for this comment. This cross-sectional study was part of the baseline assessment of a longitudinal cohort study that targeted depressed pregnant women for intervention.  The non-depressed women were not included in this study because the study interest was to understand the experiences of depression pregnant women to provide a way forward for an intervention study. Therefore we were interested in depressed pregnant women only, and it was necessary to find out their experiences for depression to get the starting point for the intervention. That’s why non-depressed women were not included.  However, I agree that including non-depressed women could be an advantage to know their experiences too.  See FDGS section.

 3. Eligibility criteria in this study were only age and pregnancy. What criteria made participants not eligible for the study? Which confounding factors were considered?

**Response:** - Thank you for this comment. The eligibility criteria were pregnant women who are depressed. As explained in the comment above, this study was part of the baseline data of an intervention study that targeted women of reproductive age and who are depressed so that they can be considered for f intervention. So it included women who gave written informed consent and women who are not mentally disturbed to the extent of not giving correct information.  See method 

 4. Which quality assurance measures were put in place for qualitative data analysis?

**Responses:** - We used the COREQ (Consolidated criteria for Reporting Qualitative research) Checklist, a 32-item checklist for interviews and focus groups.  Developed from: Tong A, Sainsbury P, Craig J. Consolidated criteria for reporting qualitative research (COREQ).

 A checklist of items was included to report this qualitative research.  Each of the items listed in this checklist was considered in the manuscript and was reported. The completed checklist was uploaded as part of my submission with this manuscript. See FGDs section

 5. Ethically; what happened to women found to have depression and especially those who had suicidal ideation as well as those experiencing violence?

**Responses: - **This was revised and added to the text, All women who appeared to have suicidal ideation and those who were experiencing violence were referred to the mental health services available at the health facility for treatment, furthermore, the second part of the study was an intervention study so they were enrolled to proceed with the intervention study, See results depression levels.

**6. Please check the following:**

 i. Age range in the text is 18-44 while in the tables it is 18-42?

**Responses: - **The actual age range of women recruited and their data analyzed had an age range of 18-42. See results, sociodemographic characteristics

 ii. Table 1: What does the percentage of the mean age represent? 

**Response:** - This was a mistake and the mistake has been corrected. See table 1

 iii. Parity is equated to the number of children below 18 years; this is erroneous, a 44 can have children above 18 and therefore be misclassified?

**Response: -** Thank you for this comment. The age range of women recruited and their data analyzed in the study was 18 to 42. None of them had a child above 18. As explained above the inclusion criteria has been corrected included women aged range of 15-49, but the actual age range of women obtained in the study was 18 - 42. Therefore there was no mix-up, and the age range here was not equated with a number of children below 18. See results.

 iv. In the table the bands for income overlap, check. Confirm the proportion of those earning less than 1500?

**Response: -** This has been revised and the mistake and has been corrected in the table.

 v. Reported range of depression score conflict some sections 0-30, others 0-26?

**Response: -** This has been clarified, Edinburgh Postnatal Depression Scale (EPDS **), **guided the scoring system ^17^ .  EPDS has 10 questions which generate scores from 0 to 30 maximum. According to this tool, a score of 10/30 or greater indicates possible depression. Women who score above 13/30 are likely to be suffering from clinical depression of varying severity.  On the results, the actual scores of women screened in the study and their data analyzed had EPDS scores range of 0 -26. See results prevalence of Prepartum depression

 vi. Table 2; confirm the totals (N) for depressed and non-depressed? 

**Response:** - Thank you for this comment; Table 2 has been revised, the labeling error for table 2 has been corrected, the number of depressed and not depressed has been confirmed. See table3

 vii. Table 3 categorize per site?

**Response: -** Table 3 has been revised and categorized per site, where the first table is depressed women from Kawangware and the second table is depressed women from Kangemi.  See table 3.

 viii. The qualitative data need to be reworked and triangulated appropriately with the quantitative data. The themes, for example, poverty, and financial struggles are closely related and therefore need to see which quotes fit well in each theme?

**Response: -** Thank you for this comment. The themes were only four. Poverty is one theme that was – manifested through three sub-themes identified as financial struggle, unemployment, and food insecurity, so financial struggles were the sub-theme of poverty, not a different theme. The other theme was Social support: Marital disharmony and Trauma experiences and all have sub-themes, and their experiences were discussed in details in the quotes See qualitative results

 ix. Review the language in the quotes and generally in the qualitative section? 

**Responses: -** I have revised my best possible the quotes in the qualitative section

 x. Discussion section; a good discussion but can be improved by focusing on the audience and key message built from the major findings. Check findings from other countries, regions, and local which used a similar and different methodology, and if possible similar context?

**Response:-**Thank you. The discussion section has been revised. I have tried to improve it as suggested. See discussion   

                     

 xi. Discuss some more weaknesses/limitations that the cross-sectional nature of the study therefore not able to establish a causal relationship, not having assessed other possible contribution to depression and neonatal outcomes?

**Responses **Thank you for this comment. I have discussed more weakness/ limitations of the study and added to the text / other possible confounding factors such as child preference, previous history of depression, spouse alcohol intake as a risk factor for depression and neonatal outcomes were not assessed hence limitation to this study. Moreover, this was a cross-sectional study and as a limitation, cross-sectional studies do not establish the causal relationship hence the limitation for this study. See study limitation.
